# Experimental and Theoretical Force Constants as Meaningful Indicator for Interatomic Bonding Characteristics and the Specific Case of Elemental Antimony

**DOI:** 10.1002/adma.202416320

**Published:** 2025-01-02

**Authors:** Franziska Zahn, Jan Hempelmann, Christopher Benndorf, Hans H. Falk, Konrad Ritter, Sergiu Levcenko, Edmund Welter, Oliver Oeckler, Richard Dronskowski, Claudia S. Schnohr

**Affiliations:** ^1^ Felix‐Bloch‐Institut für Festkörperphysik Universität Leipzig Linnéstraße 5 04103 Leipzig Germany; ^2^ Laboratory for Materials and Structures Institute of Science Tokyo Yokohama 226−8503 Japan; ^3^ Institute of Inorganic Chemistry RWTH Aachen University 52074 Aachen Germany; ^4^ Institute of Inorganic Chemistry and Crystallography Johannisallee 29 04103 Leipzig Germany; ^5^ Deutsches Elektronen‐Synchrotron DESY Notkestraße 85 22607 Hamburg Germany

**Keywords:** antimony, chemical bonding, density functional theory, force constants, X‐ray absorption spectroscopy

## Abstract

Stable Sb exhibits a rhombohedral structure, often referred to as distorted primitive cubic, with each Sb atom having three short and three longer first neighbor bonds. However, this crystal structure can also be interpreted as being layered, putting emphasis on only three short first neighbor bonds. Therefore, temperature‐dependent extended X‐ray absorption fine structure (EXAFS) spectroscopy is carried out at the Sb K‐edge in order to obtain more detailed information on local structural and vibrational properties. Evaluation of the temperature‐dependent bond lengths provides the temperature‐dependent Peierls distortion while the temperature dependence of the variance of the interatomic distance distribution yields the EXAFS force constants. Ab initio density functional theory (DFT) calculations are used for determining projected force constants. Both EXAFS and DFT force constants are compared to those of other materials with different bonding characteristics, including two‐center covalently bonded semiconductors, multicenter bonded IV–VI and V_2_VI_3_ compounds, and metallic Cu. Clearly, Sb exhibits characteristics of both localized covalent bonding and delocalized multicenter bonding. This suggests a continuous transition between these two bonding scenarios and adds to the understanding of bonding in elemental Sb in particular and in IV–VI and V_2_VI_3_ materials in general.

## Graphical abstract

The study investigates experimental and theoretical force constants of antimony. Comparison with other materials indicates a continuous transition from multicenter bonding to covalent bonding characteristics. This makes the force constants a clear indicator for the degree of multicenter bonding in electron‐rich materials and a fingerprint in material screening for future phase change material or thermoelectric applications.

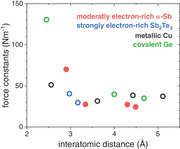

## 1 Introduction

**Figure 1:**
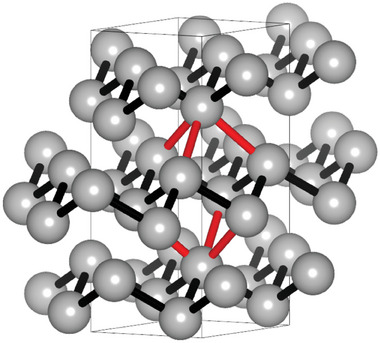
Crystal structure of stable α‐Sb ($R\;\bar{3}\;2/m$).^[^ ^18^ ^]^ Image created using.^[^ ^19^ ^]^

Antimony‐containing materials have increasingly drawn scientific interest over the last years due to incorporation in various state‐of‐the‐art technological applications:^[^ ^1^, ^2^, ^3^, ^4^ ^]^ Antimony chalcogenides are being studied for their usability as more efficient materials for solar cells and are already being employed in organic‐inorganic hybrid solar cells.^[^ ^5^, ^6^ ^]^ Sb can also be found in thermoelectrics^[^ ^1^, ^7^, ^8^ ^]^ as well as phase change memory devices made of ternary Ge‐Sb‐Te (GST) phase change materials (PCMs).^[^ ^2^, ^3^, ^6^, ^9^, ^10^, ^11^, ^12^, ^13^ ^]^ In order to tailor the material properties to the desired application, it is necessary to understand their origin. As a main‐group V (group 15) element, Sb has five valence‐electrons per lattice site, i.e., one more than materials fulfilling the simple octet rule, and is hence denoted as “electron‐rich”, similar to IV–VI and V_2_VI_3_ materials. The bonding of such electron‐rich materials is discussed in detail in Refs.^[^ ^14^, ^15^, ^16^ ^]^ Antimony exhibits a stable equilibrium rhombohedral structure ($R\bar{\;3}\;2/m$) and several metastable crystalline phases at high temperature and/or high pressure.^[^ ^17^ ^]^ The crystal structure of stable α‐Sb is shown in **Figure** **1**,^[^ ^18^, ^19^ ^]^ emphasizing the rhombohedral structure, often also referred to as distorted primitive cubic crystal structure with a coordination number of *N* = 3 + 3 = 6. This is indicated by the combination of three 1^st^ nearest neighbor (NN) bonds (black) and three 2^nd^ NN bonds (red), such that the distorted octahedral coordination does not observe the 8−*N* rule. Figure 1 also supports an alternative interpretation as a layered crystal structure with only three intralayer 1^st^ NN bonds per Sb atom corresponding to coordination of *N* = 3, which would fulfill the 8−*N* rule. Since there is a strong correlation between crystal structure, bonding characteristics, and material properties, it is desirable to investigate the bonding of elemental Sb in detail. Therefore, the local structure and vibrational properties of α‐Sb were studied using temperature‐dependent extended X‐ray absorption fine structure (EXAFS) measurements to gain a better understanding of the bonding in elemental Sb.

For this purpose, the behavior of the experimentally determined 1^st^, 2^nd^, 3^rd^, and 4^th^ NN force constants of α‐Sb is analyzed by discussing the bond length dependence of force constants reported in the literature^[^ ^20^, ^21^, ^22^, ^23^, ^24^ ^]^ and by comparing it to EAXFS force constants of materials with well‐established types of bonding.^[^ ^7^, ^25^, ^26^, ^27^, ^28^, ^29^, ^30^, ^31^, ^32^ ^]^ Furthermore, projected force constants were calculated using ab initio density functional theory (DFT), which is commonly used to study electronic properties and bonding characteristics in PCMs, thermoelectrics, and other chalcogenides theoretically.^[^ ^6^, ^10^ ^]^ Calculations were performed for stable α‐Sb and metastable simple cubic β‐Sb ($Pm\bar{3}m$)^[^ ^17^ ^]^ to further characterize the bonding characteristics arising from different crystal structures. The results are compared to prior studies^[^ ^10^, ^11^ ^]^ in which the bonding mechanism of electron‐rich GeTe was investigated based on DFT calculations for stable α‐GeTe, exhibiting rhombohedral, i.e., distorted rock‐salt structure (*R*3*m*), comparable to α‐Sb, and metastable cubic β‐GeTe, exhibiting a regular rock‐salt type structure ($Fm\bar{3}m$) similar to β‐Sb.

## 2 X‐Ray Absorption Spectroscopy Study of α‐Sb

### 2.1 Measurements and Data Analysis

Commercial antimony powder with the thermodynamically stable α‐phase and a purity of 99.999% (Onyxmet, Poland) was milled in a ball mill together with ultra‐high purity graphite (99.999%, Alfa Aesar, Germany). Subsequently, the antimony‐graphite mixture was pressed into 1 mm thick pellets with a diameter of 5 mm. This approach optimizes sample handling as well as mechanical stability. The amount of α‐Sb powder contained in the sample pellets was adjusted for optimum signal‐to‐noise ratio at the Sb K‐edge.

**Figure 2:**
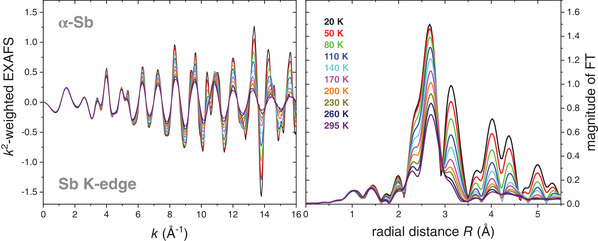
(left) *k* ^*2*^‐weighted EXAFS in k‐space and (right) magnitude of the Fourier transform (FT) in radial space for α‐Sb measured at ten different temperatures from 20 to 295 K. The amplitude of the signal strongly decreases with increasing temperature. Furthermore, three clearly separated groups of maxima can be identified in the FTs.

EXAFS measurements were performed in transmission mode at the Sb K‐edge (30 491 eV) at beamline P65 of PETRA III at DESY, Germany.^[^ ^33^ ^]^ The measurements were carried out at ten different temperatures ranging from 20 to 295 K using a liquid helium cryostat with temperature stability of better than 1 K. For temperatures *T* = 20, 230, 260, 295 K several spectra were recorded and analyzed individually to confirm the reproducibility of the determination of structural parameters. In addition, spectra of an Sb foil (beamline P65), kept at room temperature, were measured simultaneously with the sample pellet for the purpose of energy alignment. Data processing was performed using ATHENA^[^ ^34^ ^]^ based on the IFEFFIT code.^[^ ^35^ ^]^ For all spectra measured, the *k*‐window for the Fourier transformation was chosen as (3–14.5) Å^−1^ and the tapering parameter was set to 2 Å^−1^. **Figure** **2** shows the *k^2^*‐weighted EXAFS spectra in *k*‐space up to 16 Å^−1^ (left) and the magnitude of the Fourier transform (FT) in radial space up to 5.5 Å (right) for the whole temperature series. In both graphs, the amplitude of the spectra decreases with increasing temperature. Nevertheless, there is a significant signal for the entire temperature series up to 5 Å, see Figure 2 (right), and basically no noise up to 16 Å^−1^, compare with Figure 2 (left), proving a very high data quality.

**Figure 3:**
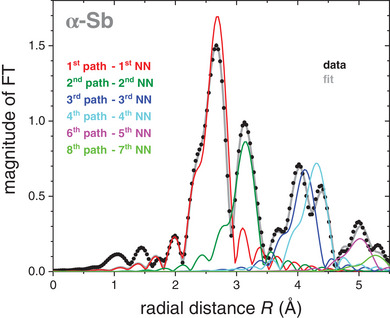
EXAFS data of α‐Sb measured at 20 K as the magnitude of the FT versus radial distance *R* (black symbols) together with the respective fit (grey line) and the individual contributions of each scattering path of the chosen fit model (colored lines).

The fitting procedure was carried out with the software package LARCH^[^ ^36^ ^]^ with scattering amplitudes and phase shifts computed by FEFF9.^[^ ^37^ ^]^ The fit was performed in radial space over a range of (1.9–5.5) Å with multiple *k*‐weights of (2, 3, 4). The fitting model, applied to all spectra of the temperature series, only consists of single scattering paths up to a radial distance of 5.5 Å and with a significant amplitude of at least 10%, which applies to the 1^st^ to 4^th^, 6^th^, and 8^th^ path, as shown in **Figure** **3**. These scattering paths correspond to the 1^st^ to 5^th^ and 7^th^ NN. The 5^th^ path is a double scattering path and the 7^th^ path is a single scattering path with less than 10% amplitude. No higher scattering paths were integrated into the fitting model, as there is no longer sufficient signal with increasing temperature at higher radial distances. For all spectra, the difference of the threshold energy *ΔE_0_* and the amplitude reduction factor $S_{0}^{2}$ were set to fixed values of –0.6 eV and 0.95, respectively, determined to be the best‐fitting values for the entire data set. Each path was assigned its own variable for the average interatomic distance *d* (bond length) and the variance *σ^2^* (mean squared relative displacement) as fitting parameters. The third cumulant *C_3_* describes the asymmetry of the distribution and is therefore highly correlated with the average distance *d*. It was included for the 1^st^ and 2^nd^ NN paths and was set to a fixed value for each temperature, the determination of which will be discussed below. For the remaining four paths, the third cumulant was set to be zero, because the strong overlap of the scattering paths (see Figure 3) and the low amplitude of the signal for higher temperatures (see Figure 2) did not allow a reasonable inclusion. Since considering *C_3_* is necessary to obtain precise distance values, the distance *d* is discussed only for the 1^st^ and 2^nd^ NN path. For the 3^rd^ and 4^th^ NN path, the discussion is limited to *σ^2^*. The 6^th^ and 8^th^ scattering path were taken into account since they show non‐negligible contributions in the range of the 3^rd^ and 4^th^ NN path,^[^ ^38^ ^]^ however, their corresponding fit parameters were not analyzed any further. The influence of the settings for *k*‐ and *R*‐space windows, of the choice of *E_0_* and $S_{0}^{2}$ and of other parameters of the data analysis on the fitting results was evaluated by systematically varying them. The resulting changes are smaller than the fit uncertainties and therefore require no additional consideration.

The temperature dependence of the structural parameters is analyzed in the framework of the correlated Einstein model. In general, the effect of atomic displacements is described by the so‐called Debye‐Waller factor. In crystallographic structure analysis, this refers to displacements of atoms from their crystallographic lattice sites,^[^ ^39^, ^40^ ^]^ whereas in X‐ray absorption spectroscopy *σ^2^* corresponds to the change in relative distance between a pair of atoms.^[^ ^41^ ^]^ The temperature dependence of both the crystallographic and the EXAFS Debye‐Waller factor can be characterized using the Einstein or the Debye model.^[^ ^40^, ^41^ ^]^ While the Debye model is primarily used for Bravais crystals, the Einstein model is more suitable for non‐Bravais crystals,^[^ ^41^ ^]^ such as antimony. In the Einstein model, the phonon spectrum is approximated by a single frequency treating either an atom (crystallographic structure analysis)^[^ ^39^, ^40^ ^]^ or a pair of atoms (EXAFS) as a harmonic oscillator with a fixed frequency.^[^ ^41^, ^42^ ^]^ In the case of EXAFS, the fixed frequency can also be interpreted as an effective force constant for the relative displacement of the atoms, including both the direct interaction between the pair of atoms and the effects of the surrounding matrix.^[^ ^43^ ^]^ The Einstein model typically yields different frequencies or effective force constants for the different coordination shells.^[^ ^42^ ^]^ Anharmonic contributions to the atomic pair potential can be accounted for by extending the initial harmonic approximation to the third cumulant *C_3_*.^[^ ^42^, ^44^ ^]^

### 2.2 Structural Parameters and Force Constants

**Figure 4:**
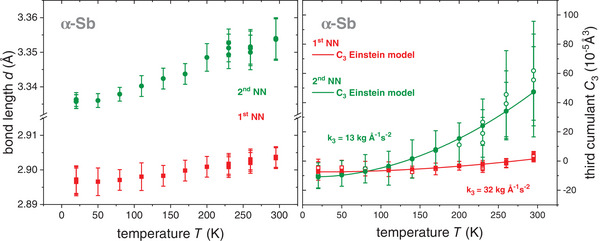
(left) Bond lengths *d* and (right) asymmetry parameter *C*_*3*_ of the 1^st^ NN (red) and 2^nd^ NN (green) of α‐Sb versus the temperature *T*. For both parameters, the values increase with increasing temperature as expected. The *C*_*3*_ values (right) are fitted with the Einstein model (Equation 1) and the values provided by this fit are indicated by full symbols. They were fixed for a repetition of the fitting procedure of the EXAFS spectra in order to reduce the correlation of the fitting parameters. The resulting bond lengths for the 1^st^ and 2^nd^ NN are shown on the left. Different data points at temperatures *T* = 20, 230, 260, and 295 K correspond to different scans. Clearly, the values match very well with their uncertainty.

The temperature‐dependent interatomic distance *d* for the 1^st^ and 2^nd^ NN (bond length) of α‐Sb as well as the corresponding asymmetry parameter *C_3_* resulting from the EXAFS measurements are plotted in **Figure** **4**. Initially, the spectra were fitted with *d* and *C_3_* as free variables, see empty symbols in Figure 4 (right). The temperature dependence of *C_3_* can be approximated with the Einstein model via the Einstein temperature *θ_E_*, the cubic force constant *k_3,_* and the static contribution to the asymmetry parameter *C_3,S_*:^[^ ^28^ ^]^

(1) $$C_{3}\left(T\right)=\frac{\hbar^{6}k_{3}}{\mu^{3}{k_{B}}^{4}{\theta_{E}}^{4}}\left\{\frac{3}{2}{\left[\coth\left(\frac{\theta_{E}}{2T}\right)\right]}^{2}-1\right\}+C_{3,S}$$

Here ℏ describes the reduced Planck constant, *k_B_* is Boltzmann's constant, and *μ* denotes the reduced mass of the absorbing and scattering atom. The Einstein temperature *θ_E_* is obtained from the temperature dependence of the variance *σ^2^*, as described below, leaving *k_3_* and *C_3,S_* as free parameters. The *C_3_(T)* values resulting from the fit with Equation (1), see full symbols in Figure 4 (right), were then set as fixed parameters and the fit of the EXAFS spectra was performed again. This leads to a reduction of the correlation and hence variation of *d* and the resulting temperature‐dependent values for 1^st^ and 2^nd^ NN are depicted on the left of Figure 4. As expected, the bond lengths increase with increasing temperature, reaching 2.904 ± 0.003 and 3.354 ± 0.006 Å for 1^st^ and 2^nd^ NN at room temperature, comparing favorably with the literature and X‐ray.^[^ ^18^ ^]^ For the 1^st^ NN, the increase amounts to 0.01 Å when going from 20 K to room temperature. This is similar to the bond length increase determined by EXAFS for Ge,^[^ ^25^, ^45^ ^]^ GaAs,^[^ ^32^ ^]^ InP,^[^ ^28^ ^]^ CdTe^[^ ^31^ ^]^ and Cu.^[^ ^27^ ^]^ For the 2^nd^ NN, the increase of the bond length within increasing temperature is slightly more pronounced and amounts to 0.02 Å. The values for the anharmonic constant *k_3_* resulting from fitting the Einstein model to the temperature‐dependent *C_3_* data of α‐Sb are 32 ± 4 and 13 ± 1 kgÅ^−1^s^−2^ for 1^st^ and 2^nd^ NN, respectively, a similar order of magnitude as the first shell results of CdTe^[^ ^31^ ^]^ and Cu.^[^ ^27^ ^]^

**Figure 5:**
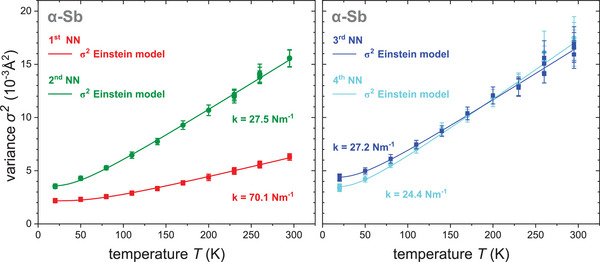
Mean squared relative displacement *σ* ^*2*^ (left) for the 1^st^ and 2^nd^ NN and (right) for the 3^rd^ and 4^th^ NN versus temperature *T* together with the corresponding fit using the Einstein model (Equation 2). The experimental values of all four neighbors are described very well by the Einstein model. Different data points at temperatures *T* = 20, 230, 260, and 295 K correspond to different scans. Clearly, the values match very well with their uncertainty.

The measurements also yield the variance *σ^2^* of the interatomic distance distribution as a function of temperature, ranging from 20 K to room temperature. The data are shown for the 1^st^ and 2^nd^ NN in **Figure** **5** on the left and for the 3^rd^ and 4^th^ NN on the right. Figure 5 clearly shows that *σ^2^* increases with increasing temperature *T*. The values for the 2^nd^ NN increase noticeably stronger compared to those of the 1^st^ NN, whereas for the 3^rd^ and 4^th^ NN *σ^2^* increases similarly with temperature as for the 2^nd^ NN. The temperature dependence of *σ^2^* can also be described by the Einstein model with $\sigma_{S}^{2}$ being the static disorder contribution^[^ ^28^, ^46^ ^]^

(2) $$\sigma^{2}\left(T\right)=\frac{\hbar^{2}}{2\mu k_{B}}\frac{1}{\theta_{E}}\cdot \coth\left(\frac{\theta_{E}}{2T}\right)+{\sigma_{S}}^{2}$$

The fits of the experimental data with the Einstein model are also shown in Figure 5, yielding an excellent agreement of experimental values and the fit. The resulting Einstein temperature *θ_E_* can be converted into the effective EXAFS force constant *k* via the Einstein frequency *ω_E_* by *ω_E_ = k_B_ θ_E_ ℏ* ^−1^ and thus $k=\mu \;\omega_{E}^{2}=\mu \frac{\theta_{E}^{2}k_{B}^{2}}{\hbar^{2}}$. The Einstein temperature, the force constant, and the static contribution for all four neighbors are listed in **Table** **1**. The Einstein temperature *θ_E_* and therefore the corresponding force constant *k* for the 1^st^ NN is much higher than those of the following three neighbors, corresponding to a factor of about three in terms of force constants. The force constants for the 2^nd^ to 4^th^ NN are of the same order of magnitude and only vary slightly amongst each other. This reflects the difference that can also be seen in the increase with temperature in the experimental data in Figure 5.

**Table 1:** Results of the fit of the temperature‐dependent variance *σ^2^* with the Einstein model (Equation 2). The fit provides the Einstein temperature *θ_E_*, which can be converted into the EXAFS force constant *k*, as well as the static disorder $\sigma_{S}^{2}$ for 1^st^ to 4^th^ NN.

NN	θ_*E*_ [K]	*k* [Nm^−1^]	$\sigma_{S}^{2}$ [10^−3^Å^2^]
1	201.2 ± 0.7	70.1 ± 0.5	0.19 ± 0.003
2	126.0 ± 0.5	27.5 ± 0.2	0.41 ± 0.04
3	125.3 ± 0.7	27.2 ± 0.3	1.20 ± 0.06
4	118.7 ± 0.8	24.4 ± 0.3	0.12 ± 0.08

In comparison, the static disorder $\sigma_{S}^{2}$ shows a rather different behavior. The 3^rd^ NN clearly yields the highest value among the neighbors evaluated in this work. It is ten times larger than the smallest $\sigma_{S}^{2}$ value, which occurs for the 4^th^ NN. The static disorder $\sigma_{S}^{2}$ for the 1^st^ NN, 2^nd^ NN, and 4^th^ NN are of a comparable order of magnitude, as can be taken from Table 1.

## 3 Density Functional Theory Calculations of α‐Sb and β‐Sb

### 3.1 Methodology

The projected force constants were calculated from density‐functional theory (DFT) using VASP^[^ ^47^, ^48^, ^49^, ^50^ ^]^ and Phonopy.^[^ ^51^ ^]^ The simulations were run using projector‐augmented waves (PAW),^[^ ^52^ ^]^ a kinetic energy cutoff of 500 eV, the solid state‐optimized GGA functional PBEsol^[^ ^53^ ^]^ and a D3‐correction term with Becke‐Johnson damping^[^ ^54^, ^55^ ^]^ with convergence criteria of 10^−8^ eV for electronic and 5 × 10^−3^ eV Å^−1^ for ionic steps. A *k*‐point mesh density of ≈0.05 Å^−1^ was used to sample the Brillouin zones.

After structural optimization, the phononic finite‐displacement calculations were run at the **Γ**‐point using 5 × 5 × 2 and 10 × 10 × 10 supercells for the α‐ and β‐Sb structures, respectively. The Phonopy‐generated pairwise force constant matrices were then projected along the interatomic vectors following the procedure outlined in reference^[^ ^10^ ^]^ to receive the projected force constants.

### 3.2 Projected Force Constants

**Figure 6:**
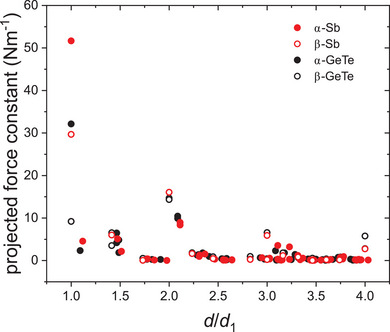
DFT projected force constants for Sb (this study) and GeTe.^[^ ^10^ ^]^ The force constants are shown as full symbols for the α‐phase and as open symbols for the β‐phase as a function of the interatomic distance *d* relative to the 1^st^ NN bond length *d*_1_.

**Figure** **6** shows DFT force constants of α‐ and β‐Sb (this study) and α‐ and β‐GeTe.^[^ ^10^ ^]^ GeTe nicely serves for comparison because its average valence electron concentration (5) equals the one of Sb. When comparing α‐Sb and α‐GeTe, which is justified because their crystal structures are similar, the largest force constant values occur for the 1^st^ NN bond. The 1^st^ NN force constant of α‐Sb (≈52 Nm^−1^), however, is larger than the corresponding 1^st^ NN force constant of α‐GeTe (≈32 Nm^−1^). Both α‐Sb and α‐GeTe exhibit a strong decrease of the DFT force constant from 1^st^ to 2^nd^ NN by roughly one order of magnitude, the ratios being 11.4 and 13.7, respectively. This trend of the shorter bond being stiffer than the longer bond has been observed before.^[^ ^10^ ^]^ For higher NN, the force constants of α‐Sb and α‐GeTe also show very similar behavior with increasing interatomic distance. They only vary slightly and are consistently very low, not exceeding ≈10 Nm^−1^. At an interatomic distance of roughly twice the 1^st^ NN distance (*d/d*_1_ ∼ 2), α‐GeTe and α‐Sb exhibit a larger force constant compared to all other higher NN depicted in Figure 6. For α‐GeTe this increase of the force constant at ≈2*d*_1_, i.e., at ∼ 1*a* with *a* being the lattice parameter, was attributed to long‐range interactions arising from multicenter bonding.^[^ ^10^ ^]^

The behavior of the DFT force constants of the more regular β‐Sb and β‐GeTe structures with perfectly octahedral coordination is different from those of the corresponding α‐phases. In contrast to α‐Sb, α‐GeTe, and β‐GeTe, the absolute values of the force constants calculated for β‐Sb somewhat depend on the size of the supercell used. Therefore, only the qualitative behavior of the force constants with increasing interatomic distance is discussed. The 1^st^ NN force constant of β‐Sb is again larger than the 1^st^ NN force constant of β‐GeTe. Both materials exhibit a decrease in force constant from 1^st^ to 2^nd^ NN, which corresponds to a ratio of ≈5 (β‐Sb) and ≈1.4 (β‐GeTe). No monotonic decrease in DFT force constant can be observed with increasing interatomic distance for β‐Sb and β‐GeTe. Instead, their force constants decrease continuously except for interatomic distances representing multiples of the 1st NN bond length *d*_1_ (i.e., 0.5*a*, 1*a*, 1.5*a*, 2*a*), for which they increase significantly due to pronounced long‐range interactions.^[^ ^10^ ^]^ Each increase is followed by a decrease in force constant until the interatomic distance matches once more a multiple of *d*_1_. Most strikingly, for β‐GeTe, the 1^st^ NN bond does not yield the largest force constant but is instead exceeded by the force constant corresponding to the interatomic distance of 2*d*_1_ ∼ 1*a* due to long‐range interactions of strong directional isotropy.^[^ ^10^ ^]^

Therefore, it can be summarized that isoelectronic Sb and GeTe exhibit strong similarity for each given phase although there are clear differences between the DFT force constants of the α‐ and β‐structure for each material. For the α‐phases with 3 + 3 coordination, a significant decrease in force constant from 1^st^ to 2^nd^ NN reflects the bond length difference between the shorter and longer bonds. For the β‐phases with perfect octahedral coordination, the overall decrease is considerably weaker. For higher NN, force constants of the α‐phase are consistently low and only vary slightly, whereas the force constants of the β‐phase exhibit pronounced maxima for interatomic distances matching multiples of the 1^st^ NN bond length *d*_1_. Based on this comparison and prior findings,^[^ ^10^ ^]^ it can be concluded that for both Sb and GeTe the characteristics of two‐center covalent bonding are more pronounced in the α‐phases, even though long‐range interactions can also be identified. Regarding the structurally regular, highly symmetric β‐phases, long‐range multicenter bonding is clearly dominant.

## 4 Discussion

**Figure 7:**
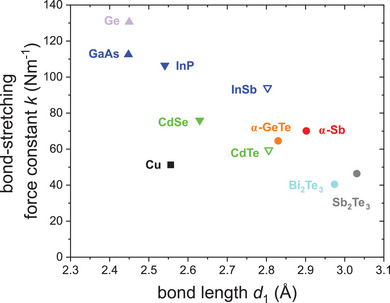
First NN EXAFS bond‐stretching force constants as a function of bond length for electron‐rich α‐Sb and other elemental materials and compounds, namely Cu,^[^ ^27^ ^]^ Ge,^[^ ^25^ ^]^ GaAs,^[^ ^32^ ^]^ InP,^[^ ^28^ ^]^ InSb,^[^ ^30^ ^]^ CdTe,^[^ ^31^ ^]^ CdSe,^[^ ^26^ ^]^ α‐GeTe,^[^ ^29^ ^]^ Sb_2_Te_3_ and Bi_2_Te_3_.^[^ ^7^ ^]^

**Figure** **7** shows the 1^st^ NN EXAFS force constant (bond‐stretching force constant) of α‐Sb together with the corresponding force constants for other elements and compounds reported by literature.^[^ ^7^, ^25^, ^26^, ^27^, ^28^, ^29^, ^30^, ^31^, ^32^ ^]^ All force constants were determined from temperature‐dependent EXAFS measurements using the Einstein model (Equation 2), hence they can be directly compared. It is possible to group these materials into three categories: i) materials fulfilling the simple octet rule and characterized by tetrahedral coordination with regular 2c‐2e (two center–two electron) covalent or polar‐covalent bonding (Ge, GaAs, InP, InSb, CdSe, CdTe), ii) electron‐rich material systems with zero or small ionicity and significant multicenter bonding (Sb, GeTe, Sb_2_Te_3_, Bi_2_Te_3_) and iii) an element with metallic bonding (Cu). Among the materials with tetrahedral coordination, Ge exhibits the largest bond‐stretching force constant. At a comparable bond length, the III‐V semiconductor GaAs shows a somewhat lower force constant. The 1^st^ NN force constant for III–V semiconductors further decreases with increasing bond length (see values for InP and InSb). The same behavior can be observed for the II‐VI semiconductors CdSe and CdTe. However, the force constants are again smaller compared to III–V semiconductors at similar bond lengths (see values for InSb and CdTe). The decrease of the force constants with increasing bond length for a given group of materials (IV, III–V, or II–VI) is in good qualitative agreement with prior findings for these semiconductor compounds,^[^ ^22^, ^23^, ^24^ ^]^ crystalline elements^[^ ^21^ ^]^ and diatomic molecules.^[^ ^20^ ^]^ The trend of decreasing force constant when going from group IV to III–V to II–VI at a comparable bond length reflects the transition from pure covalent bonding to an increasingly ionic contribution to the interatomic interaction (polar‐covalent bonding). Existing force constant values for I–VII semiconductor copper halides were not taken into account, since these materials show rather different characteristics in terms of ionicity, making it difficult to compare them among themselves but also to other compounds characterized by tetrahedral coordination.^[^ ^22^, ^56^ ^]^

Both α‐Sb and α‐GeTe are electron‐rich systems, thus changing the bonding compared to exclusively 2c–2e bonded systems. Their 1^st^ NN bond‐stretching force constants are smaller than those of III–V semiconductors but slightly higher than those of II–VI semiconductors with similar bond lengths. When comparing α‐Sb and α‐GeTe, α‐Sb exhibits a slightly larger force constant despite the bond length being ≈0.07 Å longer than for α‐GeTe. This is similar to the behavior occurring for materials with tetrahedral coordination, where elemental Ge shows a higher force constant than comparable III–V compounds with similar bond length, so the increase in ionicity seems to weaken the bond‐stretching force constant. Sb_2_Te_3_ and Bi_2_Te_3_ also are electron‐rich systems but with an even higher valence electron concentration of 5.6 per atom.^[^ ^11^ ^]^ Their bond‐stretching force constants are lower than those of II–VI semiconductors, however, the bond lengths are also larger. Taking into account the decrease of the force constants with increasing bond lengths, the values of Sb_2_Te_3_ and Bi_2_Te_3_ are thus comparable to those of II–VI semiconductors. Furthermore, Bi_2_Te_3_ has a slightly higher 1^st^ NN force constant than Sb_2_Te_3_ although its bond length is slightly longer. However, the ionicity of both materials is very similar and hence the difference cannot be explained by an increased ionicity as for α‐Sb versus α‐GeTe and Ge versus GaAs. Instead, a better mutual fit of the Bi/Te valence orbitals (as expressed by their radial moments)^[^ ^57^ ^]^ may be at play in this case.

Metallic Cu exhibits the lowest 1^st^ NN bond‐stretching force constant of all elements and compounds depicted in Figure 7 when taking into account the general bond length dependence. In particular, the value is significantly smaller than the force constants of materials having a comparable bond length. This clearly demonstrates that the metallic 1^st^ NN bond is much weaker than the covalent or polar‐covalent 1^st^ NN bonds, reflecting the enormous electron deficit for bonding.

**Figure 8:**
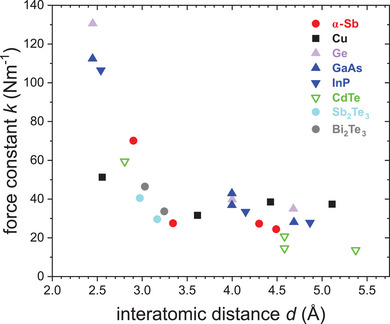
EXAFS force constants, including 1^st^ and higher NN, as a function of interatomic distance up to 5.5 Å for electron‐rich α‐Sb and other elements and compounds, namely Cu,^[^ ^27^ ^]^ Ge,^[^ ^25^ ^]^ GaAs,^[^ ^32^ ^]^ InP,^[^ ^28^ ^]^ CdTe,^[^ ^31^ ^]^ Sb_2_Te_3_ and Bi_2_Te_3_.^[^ ^7^ ^]^

The differences encountered for 1^st^ NN EXAFS bond‐stretching force constants of different material systems are also reflected in the behavior of their higher NN force constants. **Figure** **8** shows 1^st^ NN and higher neighbor force constants of Sb and other elements and compounds, again all determined from temperature‐dependent EXAFS measurements using the Einstein model (Equation 2). Figure 8 contains just parts of the materials shown in Figure 7, since only for some materials higher neighbor force constants have been reported in the literature. For all elements and compounds considered, the 1^st^ NN force constant is larger than the force constant of the 2^nd^ NN. However, the ratio of 1^st^ to 2^nd^ NN force constant varies dramatically depending on the type of material and its nature of bonding. This also applies to the overall behavior of higher neighbor force constants as discussed in the following.

The materials with tetrahedral coordination depicted in Figure 8, which all exhibit zincblende‐type (sphalerite‐type) structure, show a similar relation to that observed for 1^st^ NN bond‐stretching force constants and also for higher NN force constants. The values of elemental Ge are slightly larger than those of III‐V semiconductor compounds, which in turn have larger force constants than II–VI semiconductors, resembling the decrease in 1^st^ NN bond‐stretching force constant with increasing ionic contribution to the interatomic bonding. In absolute terms, the differences between the different compounds and elements with tetrahedral coordination are smaller for the higher NN compared to the 1^st^ NN, but the relative differences are similar. Most significantly, however, the force constants of the materials with tetrahedral coordination shown in Figure 8 exhibit a sharp drop from 1^st^ to 2^nd^ NN force constant with a ratio of ≈2.9–3.3, as summarized in **Table** **2**. Furthermore, the higher NN force constants monotonically decrease with increasing interatomic distance.

**Table 2:** Main characteristics of the materials for which EXAFS force constants are available for at least the 1^st^ and 2^nd^ NN: 1^st^ NN force constant *k*, 1^st^ to 2^nd^ NN force constant ratio, a qualitative statement on higher NN force constants, and the Peierls distortion PD. The same properties but calculated with DFT are listed for α‐Sb, α‐GeTe, β‐Sb and β‐GeTe. For β‐Sb and β‐GeTe the 1^st^ to 2^nd^ NN force constant ratio is not listed to avoid potential misinterpretations since the 2^nd^ NN of the β‐phase corresponds to the 3^rd^ NN of the α‐phase and therefore refers to completely different interatomic distances.

	Material	1^st^ NN force constant [Nm^−1^]	1^st^ to 2^nd^ NN force constant ratio	higher NN force constants	PD
EXAFS	α‐Sb	70.1	2.55	roughly constant	1.16
	Cu	51.3	1.62	roughly constant	
	Ge	130.6	3.29	monotonically decreasing	
	GaAs	112.5	3.05	monotonically decreasing	
	InP	106.5	3.18	monotonically decreasing	
	CdTe	59.4	2.88	monotonically decreasing	
	Sb_2_Te_3_	40.5	1.37	‐	1.06
	Bi_2_Te_3_	46.4	1.38	‐	1.06
DFT	α‐Sb	51.7	11.4	roughly constant	1.12
	α‐GeTe	32.1	13.7	roughly constant	1.09
	β‐Sb	29.7		roughly constant	1
	β‐GeTe	9.2		roughly constant	1

In contrast, the ratio of 1^st^ to 2^nd^ NN force constant only amounts to ≈1.6 for metallic Cu (see Table 2). Thus, the decrease is clearly less significant compared to materials crystallizing with the zincblende‐type (sphalerite‐type) structure. Furthermore, the force constants slightly increase for the 3^rd^ and 4^th^ NN compared to the 2^nd^ NN force constant, which is again strikingly different to the materials with tetrahedral coordination exhibiting a decrease in force constants with increasing interatomic distance.

Similar to the materials with tetrahedral coordination discussed before, electron‐rich α‐Sb shows a strong reduction in force constant from the 1^st^ to 2^nd^ NN, corresponding to a ratio of ≈2.5 (see Table 2). However, in comparison to the 2^nd^ NN force constant, the higher NN force constants exhibit only a minor or no decrease with increasing interatomic distance, such that 2^nd^, 3^rd^ and 4^th^ NN force constants can be considered practically constant. For the electron‐rich compounds Sb_2_Te_3_ and Bi_2_Te_3_, the reduction in force constant from 1^st^ to 2^nd^ NN is noticeably smaller compared to α‐Sb and corresponds to a ratio of only ≈1.4 for both compounds. This is similar to the ratio obtained for metallic Cu.

In summary, all materials with tetrahedral coordination exhibit a common force constant behavior. The 1^st^ NN force constant is very high for covalently bonded materials but decreases with increasing ionicity of the bond. The higher NN force constants are smaller by a factor of roughly three and slightly decrease with increasing interatomic distance. This matches very well with the strength of those bonds, arising from highly localized electrons in regular 2c–2e covalently (and polar‐covalently) bonded materials. In contrast, metallic Cu shows a definitely different force constant behavior. The 1^st^ NN force constant is very low compared to materials with a similar 1^st^ NN bond length, but also there is almost no decrease of force constants with increasing interatomic distance. As a typical metal, Cu counteracts the deficit in bonding electrons by completely delocalizing them, hence long‐ranged interactions result, which weaken the 1^st^ NN bond significantly. Very similar to Cu, Sb_2_Te_3_, and Bi_2_Te_3_ also show low 1^st^ NN force constants and only a small decrease for higher NN force constants. But in contrast to Cu, both binary compounds Sb_2_Te_3_ and Bi_2_Te_3_ are electron‐rich. However, this also leads to a weakening of the 1^st^ NN bonds due to populated antibonding levels and, even more significantly, to electronic delocalization in the form of multicenter bonds, featuring increased long‐range interactions.^[^ ^3^, ^4^, ^10^, ^11^, ^12^, ^13^, ^58^, ^59^ ^]^ Electron‐rich and electron‐deficient materials therefore exhibit a very similar behavior of their 1^st^ and higher NN EXAFS force constants. For α‐Sb, which is less electron‐rich than Sb_2_Te_3_ and Bi_2_Te_3_, the 1^st^ NN force constant is comparable to those of II–VI semiconductors with a similar bond length, and the force constants also exhibit a decrease from 1^st^ to 2^nd^ NN by a factor of ≈2.5, only slightly smaller compared to the tetrahedrally coordinated zincblende materials. Both features indicate covalent or polar‐covalent bonding. In contrast, higher NN force constants of α‐Sb only slightly decrease with increasing interatomic distance and their behavior appears intermediate to those of metallic Cu and tetrahedrally coordinated zincblende materials, indicating contributions of long‐range interaction comparable to metallic Cu in addition to short‐range 2c–2e covalent bonding.

**Figure 9:**
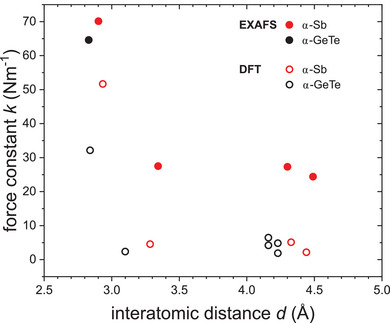
EXAFS force constants of α‐Sb (this study) and α‐GeTe^[^ ^29^ ^]^ and DFT calculated projected force constants of α‐ and β‐Sb (this study) as well as α‐ and β‐GeTe^[^ ^10^ ^]^ as a function of interatomic distance. Sb force constants are depicted in red, GeTe force constants are depicted in black. EXAFS force constants are shown as full symbols, and DFT force constants are shown as open symbols.

The similarity of the DFT‐derived force constants of α‐ and β‐Sb with α‐ and β‐GeTe, respectively, has already been discussed in Section 3.2 above, especially regarding their behavior with increasing interatomic distance. **Figure** **9** directly compares the DFT‐derived and EXAFS‐derived force constants up to an interatomic distance of 5 Å for α‐Sb and α‐GeTe^[^ ^10^, ^29^ ^]^ (see also Figures 6 and 7). This comparison is justified because both methods determine an effective force constant for the relative displacement of an atomic pair along the direction of their tie line.^[^ ^6^, ^10^, ^28^, ^43^, ^46^ ^]^ For α‐Sb, the EXAFS force constants exceed the DFT force constants by ≈20 Nm^−1^ for all NN depicted in Figure 9. Since the DFT force constants were calculated based on a harmonic approximation, a wide range of anharmonic effects were not considered. These anharmonic effects are most likely responsible for the observed deviation from the experimental values. The difference being similar for all NN suggests that these additional anharmonic forces also remain similar for all atomic pairs and do not negatively affect the quality of the comparative analysis of DFT and EXFAS force constants. A semi‐quantitative agreement can be clearly observed for their behavior with increasing interatomic distance. In particular, the DFT force constants exhibit a significant decrease from 1^st^ to 2^nd^ NN, very similar to the EXAFS force constants. For both methods, the higher NN force constants are basically constant, but also both show a slight decrease from 3^rd^ to 4^th^ NN. For α‐GeTe, no higher NN EXAFS force constants have been reported in the literature, therefore, the comparison to DFT force constants is limited to the 1^st^ NN. The behavior of these DFT force constants with increasing interatomic distance is discussed in detail in Section 3.2 above. There is an offset of ≈30 Nm^−1^ between the DFT and the EXAFS 1^st^ NN force constant of α‐GeTe, similar to α‐Sb. Moreover, for both DFT and EXAFS, α‐GeTe exhibits smaller force constants than α‐Sb. Thus, there is a clear similarity between DFT force constants and EXAFS force constants of α‐Sb and DFT force constants of α‐GeTe^[^ ^10^ ^]^ for all NN shown in Figure 9. The force constants differ in absolute values but are qualitatively similar with increasing interatomic distance. Since α‐Sb and α‐GeTe exhibit similar crystal structures and interatomic distances with the same average valence electron count (5), DFT‐derived and EXAFS‐derived force constants indicate similar contributions of electron localization and delocalization to the bonding in both materials.^[^ ^10^ ^]^

Based on the comparison of experiment and theory for Sb, GeTe, and many other materials with different bonding characteristics, it is apparent that the decrease in force constant from 1^st^ to 2^nd^ NN seen for α‐Sb indicates a mainly (2c–2e) covalent interaction, comparable to α‐GeTe.^[^ ^10^ ^]^ Nonetheless, the strength of the 1^st^ NN bond is significantly smaller and the ratio of 1^st^ to 2^nd^ NN force constant is slightly smaller than obtained for regular (2c‐2e) covalent materials like Ge or other compounds with tetrahedral coordination. The interaction hence cannot be purely short‐range covalent but also has long‐range contributions from 3c‐4e multicenter bonding due to the fact of Sb being electron‐rich, just like GeTe. The effects of long‐range interactions are also clearly visible for the higher NN force constants, leading to an almost constant magnitude of the experimentally determined and calculated force constants with increasing interatomic distance, as obtained for α‐GeTe (see Figure 9) and metallic Cu (see Figure 8), which both feature long‐range effects due to electron delocalization.^[^ ^10^, ^27^ ^]^ In the case of α‐Sb, the interaction of the three 1^st^ NN is thus dominated by localized covalent bonding (short‐range), whereas effects of delocalized 3c‐4e multicenter bonding (long‐range) are more prominent for higher NN. This is different from the hypothetical β‐phase with six 1^st^ NN of the same bond length, as the DFT calculations for β‐Sb and β‐GeTe^[^ ^10^ ^]^ indicate. The reduced 1^st^ NN force constants compared to those obtained for the α‐phase result from increased long‐ranged 3c–4e interactions, which feature delocalization and dominate 1^st^ NN bonds as well as higher NN interactions.

**Figure 10:**
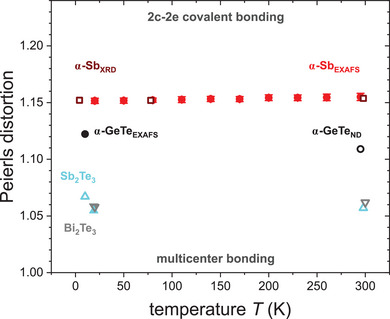
Temperature‐dependent Peierls distortion of α‐Sb at 20 to 295 K (this study), α‐GeTe at 10 K,^[^ ^29^ ^]^ Sb_2_Te_3_ and Bi_2_Te_3_ at 19 K^[^ ^7^ ^]^ derived from EXAFS 1^st^ and 2^nd^ NN bond lengths (full symbols). Peierls distortion obtained from X‐ray diffraction (XRD) for α‐Sb^[^ ^18^ ^]^ and from neutron diffraction (ND) for α‐GeTe,^[^ ^29^ ^]^ Sb_2_Te_3_ and Bi_2_Te_3_^[^ ^7^ ^]^ is shown as open symbols.

This is in line with the literature arguing that for alternating shorter and longer bonds making up near‐linear chains the degree of electron delocalization for the 1^st^ and 2^nd^ NN bond and therefore the covalent character of the bonding is connected to the relative difference of 1^st^ to 2^nd^ NN bond length.^[^ ^3^, ^6^, ^10^, ^58^, ^59^ ^]^ For the cubic β‐Sb and β‐GeTe phases, both bond lengths are the same, there is no distortion and a linear geometry of the bonding atoms provides an orthogonal alignment of p‐orbitals,^[^ ^58^ ^]^ allowing for optimum electron delocalization in the multicenter bond. In the case of rhombohedral α‐Sb and α‐GeTe phases, the 1^st^ and 2^nd^ NN bond lengths are different, which is usually referred to as (3D) Peierls distortion (PD). It describes the distortion of a metastable cubic structure with six identical bond lengths to three shorter and three longer NN bonds, thereby lowering the total energy due to weakened antibonding interactions and changing the coordination number toward fulfilling the 8–*N* rule.^[^ ^10^ ^]^ **Figure** **10** shows the Peierls distortion of α‐Sb, characterized as the ratio of 2^nd^ and 1^st^ NN bond lengths, derived from temperature‐dependent bond lengths determined by EXAFS (see Figure 4) together with literature values for α‐Sb, α‐GeTe, Sb_2_Te_3_, and Bi_2_Te_3_ determined either from EXAFS or from diffraction studies.^[^ ^7^, ^18^, ^29^ ^]^ For α‐Sb, the Peierls distortion determined using EXAFS only increases very slightly over the temperature range from 20 K to room temperature and is in excellent agreement with the corresponding values obtained by X‐ray diffraction.^[^ ^18^ ^]^ In the case of α‐GeTe, the Peierls distortion values determined by low‐temperature EXAFS and room‐temperature neutron diffraction^[^ ^29^ ^]^ are somewhat smaller than those of α‐Sb, corresponding to more similar 1^st^ and 2^nd^ NN bond lengths for α‐GeTe. Therefore, the 1^st^ NN interaction is slightly less dominated by short‐range covalent bonding and has more contributions of 3c–4e long‐range multicenter bonding, leading to a slightly reduced 1^st^ NN force constant in comparison to α‐Sb as observed in Figures 7 and 9. The correlation between Peierls distortion and bonding characteristics is further confirmed by considering the electron‐rich compounds Sb_2_Te_3_ and Bi_2_Te_3_, which feature even more similar 1^st^ and 2^nd^ NN bond lengths. This corresponds to an even less distorted crystal structure and Peierls distortion values of ≈1.06 as determined by EXAFS at 19 K and by neutron diffraction at 10 to 20 K as well as at room temperature.^[^ ^7^ ^]^ Therefore, Sb_2_Te_3_ and Bi_2_Te_3_ are expected to exhibit even stronger electron delocalization due to 3c–4e interactions than α‐Sb and α‐GeTe,^[^ ^3^, ^9^, ^10^, ^58^, ^59^ ^]^ in excellent agreement with the behavior of force constants discussed above (see Figures 7 and 8). When comparing all four electron‐rich materials discussed in the scope of this study, it thus becomes apparent that the 1^st^ NN force constant but also the ratio of 1^st^ to 2^nd^ NN force constants increase with increasing Peierls distortion, because they all increase with increasing short‐range covalent bonding character and decreasing long‐range multicenter bonding character. Interestingly, neither the force constant behavior nor the Peierls distortion seem to exhibit any gap or break, suggesting that the transition from regular (2c–2e) covalent bonding to electron‐rich multicenter bonding is continuous similar to the transition from covalent to ionic bonding.

In summary, the present study demonstrates that long‐range interactions play a decisive role in the physical properties of electron‐rich materials and are clearly reflected in the force constants and the Peierls distortion. The behavior of the force constants with increasing interatomic distance, in particular, exhibits a unique correlation with the bonding character. Experimentally or theoretically determined force constants can therefore serve as a meaningful indicator for the bonding of a specific material. Given the gradual transition between different bonding mechanisms in electron‐rich materials, this can be used to determine the degree of short‐range covalent bonding versus long‐range multicenter bonding and based thereon to estimate other material properties crucial for applications as PCM or thermoelectrics. This is especially promising with regard to high throughput theoretical material screening, where force constants could be used as fingerprints in, e.g., machine learning algorithms, to identify novel materials or to tune established materials by tailoring the level of long‐range interactions and hence the material properties.

## 5 Conclusion

Temperature‐dependent EXAFS measurements of α‐Sb were utilized to determine the 1^st^ and 2^nd^ NN bond length and the corresponding Peierls distortion as well as the force constants of the 1^st^, 2^nd^, 3^rd^, and 4^th^ NN. The 1^st^ NN bond‐stretching force constant exhibits the largest value with a significant decrease to the higher NN force constants, which are seemingly constant with increasing interatomic distance. The experimentally determined EXAFS force constants of α‐Sb were compared to those of other materials with different types of bonding reported in the literature. A large 1^st^ NN force constant and a strong decrease from 1^st^ to 2^nd^ NN force constant indicate predominantly short‐range 2c–2e covalent bonding. However, if the force constants stay constant or only vary slightly with increasing interatomic distance, long‐range effects of electron delocalization dominate. For rhombohedral α‐Sb, a pronounced drop from 1^st^ to 2^nd^ NN force constant is observed, representative of 2c–2e covalent bonding, while the behavior of the higher NN force constants indicates concomitant contributions of multicenter bonding.

In addition, projected force constants of α‐Sb and cubic β‐Sb were calculated using ab initio DFT and compared to the EXAFS force constants of α‐Sb and also to DFT force constants of α‐GeTe and β‐GeTe reported in the literature. Semiquantitative agreement between experiment and theory was observed for α‐Sb force constants, thus providing mutual confirmation for both methods. Furthermore, α‐Sb and α‐GeTe resemble each other in their DFT and EXAFS force constants, indicating similar bonding characteristics for both materials. Thus, the regular 2c–2e covalent nature of the 1^st^ NN bond as well as the electron delocalization featuring multicenter bonding for higher NN bonds is confirmed for α‐Sb. Together with the corresponding Peierls distortion values of α‐Sb and other electron‐rich materials determined from EXAFS and diffraction, these results imply a continuous transition from multicenter bonding to regular 2c–2e covalent bonding via varying contributions of different bonding characteristics. The force constant behavior is a meaningful indicator of the degree of long‐range interactions and hence crucial material properties and could be used as a fingerprint for theory‐based material screening. This detailed knowledge is key for tailoring the properties of established as well as novel materials for future PCM or thermoelectric applications.

## Conflict of Interest

The authors declare no conflict of interest.

## Data Availability

The data that support the findings of this study are available from the corresponding author upon reasonable request.

## References

[1] J. Q. Li , X. W. Feng , W. A. Sun , W. Q. Ao , F. S. Liu , Y. Du , Mater. Chem. Phys. 2008, 112, 57.

[2] S. Raoux , F. Xiong , M. Wuttig , E. Pop , MRS Bull. 2014, 39, 703.

[3] V. L. Deringer , R. Dronskowski , M. Wuttig , Adv. Funct. Mater. 2015, 25, 6343.

[4] Y. Cheng , O. Cojocaru‐Mirédin , J. Keutgen , Y. Yu , M. Küpers , M. Schumacher , P. Golub , J.‐Y. Raty , R. Dronskowski , M. Wuttig , Adv. Mater. 2019, 31, 1904316. 10.1002/adma.201904316 31489721

[5] J. A. Chang , J. H. Rhee , S. H. Im , Y. H. Lee , H. Kim , S. I. Seok , M. K. Nazeeruddin , M. Gratzel , Nano Lett. 2010, 10, 2609. 20509686 10.1021/nl101322h

[6] V. L. Deringer , R. P. Stoffel , M. Wuttig , R. Dronskowski , Chem. Sci. 2015, 6, 5255. 29449929 10.1039/c5sc00825e PMC5669248

[7] A. N. Mansour , W. Wong‐Ng , Q. Huang , W. Tang , A. Thompson , J. Sharp , J. Appl. Phys. 2014, 116, 83513.

[8] M. Cagnoni , D. Führen , M. Wuttig , Adv. Mater. 2018, 30, 1801787. 10.1002/adma.201801787 29975431

[9] B. J. Kooi , M. Wuttig , Adv. Mater. 2020, 32, 1908302. 10.1002/adma.201908302 32243014

[10] J. Hempelmann , P. C. Müller , P. M. Konze , R. P. Stoffel , S. Steinberg , R. Dronskowski , Adv. Mater. 2021, 33, 2100163. 34323316 10.1002/adma.202100163 PMC11469311

[11] J. Hempelmann , P. C. Müller , C. Ertural , R. Dronskowski , Angew. Chem., Int. Ed. 2022, 61, e202115778. 10.1002/anie.202115778 PMC9306605 35007401

[12] R. O. Jones , S. R. Elliott , R. Dronskowski , Adv. Mater. 2023, 35, 2300836. 10.1002/adma.202300836 37162226

[13] P. C. Müller , S. R. Elliott , R. Dronskowski , R. O. Jones , J. Phys.: Condens. Matter 2024, 36, 325706. 10.1088/1361-648X/ad46d6 38697198

[14] R. Nesper , Pro. Solid St. Chem. 1990, 20, 1.

[15] G. A. Papoian , R. Hoffmann , Angew. Chem., Int. Ed. 2000, 39, 2408. 10.1002/1521-3773(20000717)39:14<2408::aid-anie2408>3.0.co;2-u 10941096

[16] R. Dronskowski , Chemical Bonding: From Plane Waves via Atomic Orbitals, De Gruyter, Boston, 2023, p. 186.

[17] D. Akhtar , V. D. Vankar , T. C. Goel , K. L. Chopra , J. Mater. Sci. 1979, 14, 988.

[18] C. S. Barrett , P. Cucka , K. Haefner , Acta. Cryst. 1963, 16, 451.

[19] K. Momma , F. Izumi , J. Appl. Cryst. 2011, 44, 1272.

[20] R. M. Badger , J. Chem. Phys. 1934, 2, 128.

[21] J. Waser , L. Pauling , J. Chem. Phys. 1950, 18, 747.

[22] H. Neumann , Cryst. Res. Technol. 1985, 20, 773.

[23] H. Neumann , Cryst. Res. Technol. 1989, 24, 325.

[24] A. S. Verma , Phys. Lett. A 2008, 372, 7196.

[25] G. Dalba , P. Fornasini , M. Grazioli , F. Rocca , Phys. Rev. B Condens. Matter 1995, 52, 11034. 9980201 10.1103/physrevb.52.11034

[26] G. Dalba , P. Fornasini , R. Grisenti , D. Pasqualini , D. Diop , F. Monti , Phys. Rev. B Condens. Matter 1998, 58, 4793.

[27] P. Fornasini , S. a Beccara , G. Dalba , R. Grisenti , A. Sanson , M. Vaccari , F. Rocca , Phys. Rev. B 2004, 70, 174301. 10.1103/PhysRevLett.89.025503 12097002

[28] C. S. Schnohr , P. Kluth , L. L. Araujo , D. J. Sprouster , A. P. Byrne , G. J. Foran , M. C. Ridgway , Phys. Rev. B 2009, 79, 195203. 10.1088/0953-8984/21/15/155302 21825361

[29] P. Fons , A. V. Kolobov , M. Krbal , J. Tominaga , K. S. Andrikopoulos , S. N. Yannopoulos , G. A. Voyiatzis , T. Uruga , Phys. Rev. B 2010, 82, 155209.

[30] M. Krbal , A. V. Kolobov , B. Hyot , B. André , P. Fons , R. E. Simpson , T. Uruga , H. Tanida , J. Tominaga , J. Appl. Phys. 2010, 108, 23506.

[31] N. Abd el All , G. Dalba , D. Diop , P. Fornasini , R. Grisenti , O. Mathon , F. Rocca , B. Thiodjio Sendja , M. Vaccari , J. Phys. Condens. Matter 2012, 24, 115403. 22356832 10.1088/0953-8984/24/11/115403

[32] S. I. Ahmed , G. Aquilanti , N. Novello , L. Olivi , R. Grisenti , P. Fornasini , J. Chem. Phys. 2013, 139, 164512. 24182054 10.1063/1.4826629

[33] E. Welter , R. Chernikov , M. Herrmann , R. Nemausat , AIP Conf. Proc 2019, 2054, 40002.

[34] B. Ravel , M. Newville , J. Synchrotron Rad. 2005, 12, 537. 10.1107/S0909049505012719 15968136

[35] M. Newville , J. Synchrotron Rad. 2001, 8, 322. 10.1107/s0909049500016964 11512767

[36] M. Newville , J. Phys.: Conf. Ser. 2013, 430, 12007.

[37] J. J. Rehr , J. J. Kas , F. D. Vila , M. P. Prange , K. Jorissen , Phys. Chem. Chem. Phys. 2010, 12, 5503. 20445945 10.1039/b926434e

[38] C. S. Schnohr , L. L. Araujo , M. C. Ridgway , J. Phys. Soc. Jpn. 2014, 83, 094602.

[39] W. F. Kuhs , Acta Cryst 1992, A48, 80.

[40] A. Bentien , E. Nishibori , S. Paschen , B. B. Iversen , Phys. Rev. B 2005, 71, 144107.

[41] M. Vaccari , P. Fornasini , J. Synchrotron Rad. 2006, 13, 321. 10.1107/S0909049506018504 16799223

[42] P. Fornasini , R. Grisenti , J. Synchrotron Rad. 2015, 22, 1242. 10.1107/S1600577515010759 26289276

[43] S. Eckner , K. Ritter , P. Schöppe , E. Haubold , E. Eckner , J. Rensberg , R. Röder , M. C. Ridgway , C. S. Schnohr , Phys. Rev. B 2018, 97, 195202.

[44] T. Yokoyama , J. Synchrotron Rad. 1999, 6, 323. 10.1107/S0909049599001521 15263295

[45] G. Dalba , P. Fornasini , R. Grisenti , J. Purans , Phys. Rev. Lett. 1999, 82, 4240.

[46] C. S. Schnohr , Appl. Phys. Rev. 2015, 2, 31304.

[47] G. Kresse , J. Hafner , Phys. Rev. B Condens. Matter 1993, 47, 558. 10004490 10.1103/physrevb.47.558

[48] G. Kresse , J. Furthmüller , Comput. Mater. Sci. 1996, 6, 15.

[49] G. Kresse , J. Furthmüller , Phys. Rev. B Condens. Matter 1996, 54, 11169. 9984901 10.1103/physrevb.54.11169

[50] G. Kresse , D. Joubert , Phys. Rev. B Condens. Matter 1999, 59, 1758.

[51] A. Togo , I. Tanaka , Scr. Mater. 2015, 108, 1.

[52] P. E. Blöchl , Phys. Rev. B Condens. Matter 1994, 50, 17953. 9976227 10.1103/physrevb.50.17953

[53] G. I. Csonka , J. P. Perdew , A. Ruzsinszky , P. H. T. Philipsen , S. Lebègue , J. Paier , O. A. Vydrov , J. G. Ángyán , Phys. Rev. B 2009, 79, 155107.

[54] S. Grimme , J. Antony , S. Ehrlich , H. Krieg , J. Chem. Phys. 2010, 132, 154104. 20423165 10.1063/1.3382344

[55] S. Grimme , S. Ehrlich , L. Goerigk , J. Comput. Chem. 2011, 32, 1456. 21370243 10.1002/jcc.21759

[56] M. Vaccari , R. Grisenti , P. Fornasini , F. Rocca , A. Sanson , Phys. Rev. B 2007, 75, 184307.

[57] R. O. Jones , Phys. Rev. B 2020, 101, 24103.

[58] T. H. Lee , S. R. Elliott , Adv. Mater. 2020, 32, 2000340. 10.1002/adma.202000340 32458525

[59] F. C. Mocanu , K. Konstantinou , J. Mavračić , S. R. Elliott , Phys. Status Solidi RRL 2021, 15, 2000485.

